# Note on Anacahuite Wood, a Reputed Remedy for Consumption

**Published:** 1861-04

**Authors:** Daniel Hanbury


### *Note on Anacahuite Wood, a reputed Remedy for Consumption.* By Daniel Hanbury, F. L. S.

During the past autumn there have been several inquiries, chiefly on the part of merchants connected with Germany, for a new drug imported from Mexico under the name of *Anacahuite Wood.* A single package of this drug was offered for sale in September last by a London drug broker, and purchased for shipment to the Continent. In Germany the demand has been very considerable, and although 10,000 pounds of the wood have been imported into Bremen and Hamburg, and sold at a high rate, the requirements of purchasers are still far from being satisfied.

In order to explain the circumstances that have led to the introduction of this new drug and the valuable properties which it is asserted to possess, I will here give the translation of a short paragraph extracted from a popular German journal into the pages of the *Archiv der Pharmacie* for November last.

"There grows at Tampico in Mexico a tree with the wood of which called *Anacahuite*, the Indians cure all chest complaints, especially diseases of the lungs. The inhabitants of Tampico have also used this remedy, and have succeeded in completely curing consumption with it, even in the case of persons in whose families the disease appeared to be hereditary. The Prussian Consul at Tampico has for years past observed the beneficial effects of this wood, and, as in all cases the patients were cured by the use of it, he has been induced to communicate the subject to the Prussian Government, and to send a considerable quantity of the wood to Berlin, where experiments are now being made in the hospitals to determine its medicinal efficacy.

Anacahuite wood is administered in the simple form of infusion, shavings of the wood, previously deprived of its bark, being treated with boiling water, as in the preparation of tea. This infusion is drunk in the morning fasting and again in the evening at bedtime. In cases where the disease has already made considerable progress, the infusion may be used as often as the patient is inclined to drink. Highly seasoned food and strong alcoholic beverages as well as coffee, must be avoided while the medicine is being used. Spitting of blood is removed in a few days; in all cases, however, it is advisable to continue the use of the medicine for some time even after recovery."

Anacahuite wood, it will thus be seen, is a production of Tampico, whence, in fact, all the supplies that have reached Europe have been shipped. Its botanical origin is at present unknown. Dr. Otto Berg, who has elaborately described its external characters and anatomical structure, thinks that from the organization of the bark and wood it is probably derived from some papilionaceous tree, although there are only general appearances that guide him to such an opinion. It is to be hoped, however, that this question may be soon set at rest by good, dried specimens of the flower, fruit, and leaf of the tree being obtained from Tampico, and submitted to some competent botanical authority in Europe.

Anacahuite wood, as I have seen it, consists of truncheons of about two feet long, varying from the thickness of a finger to that of a man's arm. The wood is covered with a thick, fibrous, greyish-brown bark, coarsely furrowed longitudinally with deep cracks, and so tough that it may be stripped off in pieces of considerable length. A white, pulverulent matter, resembling an efflorescence, occurs between the layers of liber from which it escapes as dust when the bark is torn. When one examines a transverse section of a truncheon, one perceives the bark to be of considerable thickness and to consist of two more or less defined zones—the inner more compact. The wood is of a pale brown, marked with concentric zones, which, however, are too little distinguished from one another to be counted with any certainty. The pith is frequently eccentric; its transverse section sometimes shows a stellate form.

Anacahuite wood is inodorous and insipid. A strong decoction is transparent and of a sherry brown color; it is blackened by a persalt of iron, but neither a solution of gelatine nor of iodine affects it. The taste of the decoction is extremely slight and unremarkable, so that one may reasonably be permitted to doubt the extravagant, though, if true, very gratifying assertions regarding the virtues of the drug. The experiments, indeed, that have been instituted in the Great Hospital at Berlin, have hitherto had, as I am informed by Dr. C. G. Mitscherlich, no satisfactory results. The details of the cases have not, however, yet been published.—*London Pharm. Journal.*

